# Curcumin-Derived J147 Protects HT22 Cells from Cell Death Under Tunicamycin-Induced Endoplasmic Reticulum Stress with CHOP Suppression Retained After ATP5A Knockdown

**DOI:** 10.3390/cells15212005

**Published:** 2026-11-03

**Authors:** Tsugumi Tanaka, Hiroshi Nango, Komugi Tsuruta, Hiroko Miyagishi, Tadashi Saigusa, Kosuke Sugawa, Yasuhiro Kosuge

**Affiliations:** 1 Laboratory of Pharmacology, School of Pharmacy, Nihon University, 7-7-1 Narashinodai, Funabashi-shi 274-8555, Chiba, Japan; phts24004@g.nihon-u.ac.jp (T.T.); nango.hiroshi@nihon-u.ac.jp (H.N.); phko22004@g.nihon-u.ac.jp (K.T.); miyagishi.hiroko@nihon-u.ac.jp (H.M.); 2 Department of Pharmacology, School of Dentistry at Matsudo, Nihon University, 2-870-1 Sakaechonishi, Matsudo-shi 271-8587, Chiba, Japan; saigusa.tadashi@nihon-u.ac.jp; 3 Department of Materials and Applied Chemistry, College of Science and Technology, Nihon University, 1-8-14 Kanda-Surugadai, Chiyoda-ku, Tokyo 101-8308, Japan; sugawa.kosuke@nihon-u.ac.jp

**Keywords:** endoplasmic reticulum stress, C/EBP homologous protein, J147, ATP5A, oxytosis, HT22 cells

## Abstract

**Highlights:**

**What are the main findings?**

- J147 outperformed curcumin and CNB-001 against glutamate-induced oxytosis and was the only compound to protect HT22 cells from cell death under tunicamycin-induced ER stress, with reduced CHOP expression while GRP78 induction remained evident.
- ATP5A knockdown attenuated glutamate-induced oxytosis but not cell death under tunicamycin-induced ER stress.

**What are the implications of the main findings?**

- J147-mediated protection under tunicamycin-induced ER stress was retained after ATP5A knockdown and occurred in parallel with reduced CHOP induction.
- J147 may serve as a pharmacological tool to dissect oxidative versus proteotoxic stress responses in neurons and to explore CHOP-associated ER stress signaling as a target.

**Abstract:**

Oxidative stress and proteotoxic endoplasmic reticulum (ER) stress are interconnected cellular stress processes implicated in neuronal injury. J147 reportedly exerts neuroprotective effects through ATP synthase α-subunit (ATP5A)-associated mitochondrial mechanisms, but whether it protects cells from death under proteotoxic ER stress through the same mechanism remains unclear. Here, we directly compared curcumin, CNB-001, and J147 under glutamate-induced oxytosis and tunicamycin (TM)-induced ER stress in murine hippocampal HT22 cells. MTT reduction assays showed that all three compounds attenuated glutamate-induced cell death, whereas only J147 protected cells from TM-induced cytotoxicity at a submicromolar range. LIVE/DEAD staining confirmed the protective effects of J147 against both stressors. At the molecular level, J147 suppressed the TM-induced upregulation of the pro-apoptotic transcription factor C/EBP homologous protein (CHOP) at the mRNA and protein levels without suppressing the adaptive ER chaperone glucose-regulated protein 78. ATP5A knockdown attenuated glutamate-induced oxytosis but did not protect against TM-induced cell death, and J147 retained its protective effect after ATP5A knockdown. These findings support stressor-dependent protective actions of J147 involving an ATP5A-associated mechanism against oxidative stress and an ATP5A-independent mechanism associated with suppression of CHOP induction. J147 may therefore provide a useful pharmacological tool for investigating neuronal responses to interconnected oxidative and proteotoxic stress.

## 1. Introduction

Neurodegenerative diseases such as Alzheimer’s disease (AD), Parkinson’s disease (PD), and amyotrophic lateral sclerosis (ALS) are characterized by progressive neuronal dysfunction and loss, with multiple cellular stress pathways converging to promote neurodegeneration [1]. Among these pathways, oxidative stress, mitochondrial dysfunction, impaired proteostasis, and endoplasmic reticulum (ER) stress are closely interconnected contributors to neuronal injury [2,3]. Neurons are vulnerable to disturbances in ER homeostasis because of their high metabolic demand, complex morphology, and strong dependence on the ER for protein folding, lipid synthesis, calcium regulation, and intracellular trafficking [4]. Excessive accumulation of unfolded or misfolded proteins in the ER activates the unfolded protein response (UPR), an adaptive signaling network that promotes restoration of proteostasis [5]. However, excessive or prolonged ER stress shifts the UPR from an adaptive response to a pro-death program, ultimately leading to neuronal cell death [6]. These findings highlight maladaptive ER stress signaling as a promising pharmacological target for neuronal protection.

A key mediator of ER stress-induced cell death is C/EBP homologous protein (CHOP), a pro-apoptotic transcription factor induced downstream of maladaptive UPR signaling [7,8]. The causal involvement of CHOP in ER stress-induced cell death was demonstrated by the reduced susceptibility of CHOP-deficient cells to ER stress [9]. CHOP contributes to neuronal vulnerability by downregulating anti-apoptotic factors and promoting apoptotic signaling cascades under severe ER stress. Its expression is robustly upregulated in postmortem brains and experimental models of major neurodegenerative diseases, including AD [10], PD [11], and ALS [12]. Moreover, suppressing CHOP activation attenuates neuronal cell death in models of hypoxia-induced injury [13]. These studies support an important contribution of excessive CHOP activation to neuronal loss across diverse pathological contexts. Thus, selectively targeting this CHOP-associated maladaptive ER stress pathway is a potential strategy for neuroprotection.

Curcumin, a polyphenolic compound derived from *Curcuma longa*, has been extensively studied for its neuroprotective, antioxidant, and anti-inflammatory effects in experimental models of neurodegeneration [14,15,16,17]. Curcumin attenuates hypoxia-induced ER stress and CHOP expression by reducing upstream oxidative stress [18]. However, its therapeutic potential is limited by its poor chemical stability, low aqueous solubility, and restricted brain bioavailability [19]. Curcumin-derived neuroprotective compounds such as CNB-001 [20] and J147 [21] have been developed to improve potency and pharmacological properties. J147 exhibits potent neurotrophic and neuroprotective properties and improves cognitive function in animal models of brain aging and AD [22,23,24]. Preclinical studies have also demonstrated favorable bioavailability and safety profiles of J147, supporting its further development as a candidate therapeutic agent for neurological disorders [25]. ATP5A, the α-subunit of mitochondrial ATP synthase, has been identified as a molecular target of J147 and has been implicated in its effects on mitochondrial metabolism and protection against oxidative stress [26]. Because mitochondrial function, intracellular Ca^2+^ homeostasis, and ER proteostasis are closely interconnected [27], ATP5A-associated signaling could potentially influence cellular responses to ER stress. A recent in vivo study indicated that J147 attenuates ER stress-related signaling in cortical neurons after traumatic brain injury [28]. However, a direct regulatory relationship between ATP5A and CHOP induction has not been established, and it remains unclear whether J147-mediated cytoprotection and reduced CHOP induction during direct proteotoxic stress require ATP5A.

The murine hippocampal HT22 cell line, an immortalized neuronal cell line derived from mouse hippocampal cultures, exhibits neuron-like functional cholinergic properties [29]. The molecular cell death pathways in HT22 cells are highly analogous to those in primary neurons in terms of sensitivity to glutamate (Glu)-induced oxidative cell death (oxytosis) [30,31,32] and TM-induced ER stress [33,34,35]. In this model, extracellular Glu inhibits the cystine/glutamate antiporter system xc−, thereby reducing cystine uptake and glutathione synthesis and promoting ROS accumulation and oxytotic cell death [30,32]. In contrast, TM inhibits N-linked glycosylation, causing the accumulation of misfolded proteins in the ER and activation of the UPR [5,36,37]. Sustained UPR signaling can induce CHOP and contribute to cell death [7,8,9]. Thus, these two models initiate cellular injury through distinct primary mechanisms—redox imbalance in the Glu model and disruption of ER proteostasis in the TM model—allowing the effects of J147 to be compared across different stress conditions. Therefore, HT22 cells serve as a useful platform for comparing the effects of candidate neuroprotective compounds on distinct but interconnected neuronal stress pathways. Importantly, the use of a single cellular background enables direct comparison of pharmacological responses to Glu-induced oxytosis and TM-induced proteotoxic ER stress while minimizing cell-type-dependent variability. In the present study, we aimed to compare the effects of curcumin, CNB-001, and J147 on Glu-induced oxidative cell death and TM-induced ER stress-mediated cell death in HT22 cells. We further examined whether J147-mediated cytoprotection and reduced CHOP induction under TM exposure were retained after ATP5A knockdown. This design allowed us to test whether J147 engages an additional protective component distinct from its established ATP5A-associated mitochondrial action. Here, we demonstrate that J147 protects HT22 cells against chemically induced proteotoxic ER stress through an ATP5A-independent mechanism distinct from its established ATP5A-associated mitochondrial action. These findings provide a cellular framework for investigating how J147 modulates responses to distinct oxidative and proteotoxic stress conditions.

## 2. Materials and Methods

### 2.1. Reagents

TM and 3-(4,5-dimethylthiazol-2-yl)-2,5-diphenyl tetrazolium bromide (MTT) were purchased from Sigma-Aldrich (St. Louis, MO, USA). Special-grade sodium hydrogen L (+)-Glutamate monohydrate, N,N-dimethylformamide, and curcumin were purchased from Fujifilm Wako Pure Chemical Corporation (Osaka, Japan). CNB-001 and J147 were purchased from SynInnova Laboratories Inc. (Edmonton, AB, Canada) and AmBeed (Arlington Hts, IL, USA), respectively. Sodium hydrogen L (+)-Glutamate monohydrate was dissolved in purified water. Curcumin, CNB-001, J147, and TM were dissolved in dimethyl sulfoxide (DMSO) as stock solutions, and the final concentration of DMSO in the culture medium did not exceed 0.1%. Vehicle-treated cells received the same concentration of DMSO.

### 2.2. Cell Culture

HT22 cells were provided by the late Professor David Schubert from the Salk Institute for Biological Studies (La Jolla, CA, USA). The cells were maintained following protocols described in our previous work [33]. Briefly, the cells were grown in Dulbecco’s Modified Eagle Medium (Thermo Fisher Scientific Inc., Waltham, MA, USA) supplemented with 10% fetal bovine serum (Thermo Fisher Scientific Inc.) and 1% penicillin–streptomycin (Thermo Fisher Scientific Inc.). Subculturing was performed every 2–3 days. The cells were seeded at a density of 1.2 × 10^4^ cells/cm^2^. The incubation conditions were set at 37 °C in a humidified atmosphere containing 10% CO_2_. Cells were routinely tested for mycoplasma contamination and were confirmed to be negative. Cells between passages 10 and 30 were used for the experiments. After overnight attachment, the culture medium was replaced, and the cells were simultaneously exposed to Glu or TM and the indicated test compounds.

### 2.3. MTT Reduction Assay

Cell viability was assessed on the basis of cellular MTT-reducing activity as described previously [33]. Initially, HT22 cells were plated into 96-well plates (AGC Techno Glass Co., Ltd., Shizuoka, Japan) at a density of 4000 cells per well and allowed to adhere overnight. After confirming cell attachment and morphology by phase-contrast microscopy on the following day, the cells were treated with curcumin, CNB-001, or J147 in the presence or absence of Glu or TM. Concentration ranges were selected on the basis of previously reported pharmacologically active concentrations of curcumin and its derivatives, and to permit concentration–response analysis [18,20,21]. Basal effects of each compound were assessed in parallel to distinguish cytoprotection from compound-induced changes in MTT-reducing activity. Following a 24 h incubation period, cell morphology was re-evaluated, and then MTT solution was added to each well at a final concentration of 0.5 mg/mL. The plates were incubated at 37 °C for 3 h, after which the reaction was terminated by adding a solution of 50% N,N-dimethylformamide and 20% sodium dodecyl sulfate (SDS), pH 4.8. Absorbance related to formazan production was quantified the following day on a microplate reader (SH-1000Lab; Corona Electric Co., Ltd., Ibaraki, Japan) at 570 nm (reference: 655 nm). Data are presented as cell viability relative to that of the untreated group.

### 2.4. Phase-Contrast Micrographs

Morphological changes in HT22 cells were documented immediately before adding the MTT reagent. Photomicrographs were acquired using an inverted microscope (IX70; Olympus, Tokyo, Japan) with an i-NTER LENS (Microscope Network Co., Ltd., Saitama, Japan) as described previously [38]. Images were acquired using identical microscope settings across all experimental groups.

### 2.5. EC50 Calculation

Concentration–response curves were fitted using a four-parameter logistic model with a variable slope in GraphPad Prism 9.0 (GraphPad Software, San Diego, CA, USA), as described previously [39]. The half-maximal effective concentration (EC50) values and 95% confidence intervals were estimated from the fitted curves. EC50 values were considered not estimable when a well-defined maximal response was not obtained within the tested concentration range.

### 2.6. LIVE/DEAD Assay

Live and dead cells were visualized using the LIVE/DEAD^®^ Viability/Cytotoxicity Kit (Thermo Fisher Scientific Inc.) as previously described [40]. HT22 cells were seeded onto CC2-coated Lab-Tek II Chamber Slides (Nalge Nunc International, Rochester, NY, USA) and allowed to adhere overnight. Following the indicated 24 h pharmacological treatments, the cells were washed with HBSS (Thermo Fisher Scientific Inc.) and incubated with a staining solution containing 2 µM calcein-AM and 4 µM ethidium homodimer-1 (EthD-1) for 50 min at 37 °C. Images were acquired using identical settings, and quantification was performed by an investigator blinded to the treatment conditions. Quantification was performed independently by an investigator who was not involved in cell treatment. Representative images of live cells (green fluorescence) and dead cells (red fluorescence) were captured using a confocal laser scanning microscope (LSM-710; Zeiss, Oberkochen, Germany). Cell mortality was calculated as the number of EthD-1-positive cells divided by the total number of calcein-AM-positive and EthD-1-positive cells.

### 2.7. Quantitative Real-Time PCR

Quantitative real-time PCR (qPCR) was performed as described in our previous study [41]. HT22 cells were treated with test compounds in the presence or absence of TM for 3 h. In brief, total RNA was isolated using the High Pure RNA Isolation Kit (Roche Diagnostics, Basel, Switzerland). Subsequently, cDNA was synthesized from 0.5 µg of total RNA using Oligo(dT)18 primers and the Transcriptor First Strand cDNA Synthesis Kit (Roche Diagnostics). qPCR amplification was carried out using TB Green^®^ Premix Ex Taq™ II (TaKaRa, Shiga, Japan), with all reactions performed in duplicate. Relative mRNA abundance was calculated using the 2 ^−ΔΔ^CT method and normalized to that of the internal control glyceraldehyde-3-phosphate dehydrogenase (GAPDH). *Gapdh* expression was confirmed to remain stable across the experimental conditions. Primer specificity was confirmed by melting-curve analysis. The primer sequences were as follows: 5′-CCTGGTATGAGGATCTGCAGGA-3′ (forward) and 5′-AGCTAGGGACGCAGGGTCAA-3′ (reverse) for *Ddit3*; and 5′-TGTGTCCGTCGTGGATCTGA-3′ (forward) and 5′-TTGCTGTTGAAGTCGCAGGAG-3′ (reverse) for *Gapdh*.

### 2.8. Western Blot Analysis

Immunoblotting was conducted following previously established procedures [42]. HT22 cells were exposed to TM in the presence or absence of curcumin, CNB-001, or J147. For analysis of CHOP expression, cells were treated with TM in the presence or absence of the test compounds for 6 h. For analysis of glucose-regulated protein 78 kDa (GRP78) expression, cells were treated for 24 h. Densitometric analysis was performed using identical predefined settings for all samples. In brief, the lysates were resolved via electrophoresis on 7.5 or 12.5% SDS-polyacrylamide gels and subsequently transferred onto polyvinylidene difluoride membranes (Millipore, Burlington, MA, USA) over a 1 h period. Non-specific binding sites were blocked by incubating the membranes in 3% skim milk for 3 h at room temperature (15–25 °C), followed by four 10-min washes with Tris-buffered saline containing 0.05% Tween 20 (TTBS). The membranes were incubated overnight at 4 °C with the following primary antibodies: anti-CHOP (Cell Signaling Technology, cat. no. 5554, 1:1000 dilution, RRID:AB_10694399, Danvers, MA, USA), KDEL (Enzo Life Sciences, cat. no. ENZ-ABS679, 1:1000 dilution, Farmingdale, NY, USA) (used to detect the approximately 78-kDa GRP78 band), anti-ATP5A [15H4C4] (Abcam, cat. no. ab14748, 1:1000 dilution, RRID:AB_301447, Cambridge, UK), and anti-β-actin (Sigma-Aldrich, cat. no. A5441, 1:2000 dilution, RRID: AB_476744) as a loading control. After being washed five times with TTBS, the membranes were incubated with the following horseradish peroxidase-conjugated secondary antibodies for 1 h at room temperature: Amersham ECL Rabbit IgG, HRP-linked whole Ab (from donkey) (Cytiva, cat. no. NA934, 1:10,000 dilution, Marlborough, MA, USA) and Goat anti-Mouse IgG (H + L) Superclonal™ Secondary Antibody, HRP (Thermo Fisher Scientific Inc., cat. no. A28177, 1:20,000 dilution). The specificity of each primary antibody was supported by previous studies using genetic depletion or other validation approaches. Use of the anti-ATP5A [26], anti-CHOP [43], and anti-KDEL [44] antibodies in immunoblotting has been reported previously. Protein bands were visualized using an enhanced chemiluminescence system (ECL Prime; Cytiva) and captured with ImageQuant™ 800 (IQ800) (Cytiva). The optical density of the bands was quantified using ImageJ software 1.54g (National Institutes of Health, Bethesda, MD, USA), and the expression of the target proteins was normalized to that of β-actin. Western blot images were cropped and prepared using Microsoft PowerPoint (version 2019; Microsoft Corporation, Redmond, WA, USA), and contrast was adjusted using the Picture Adjustments tools in PowerPoint for better visualization.

### 2.9. Transfections

siRNA transfection was performed following previously established procedures [26]. In brief, HT22 cells were transfected with ATP5A-specific siRNA (50 nM; Santa Cruz Biotechnology, Dallas, TX, USA, sc-60228) or a non-targeting control siRNA (50 nM; Santa Cruz Biotechnology, sc-37007) using Lipofectamine RNAiMAX (Thermo Fisher Scientific Inc., cat. no. 13778) in accordance with the manufacturer’s instructions. At 24 h after transfection, the cells were exposed to Glu or TM for an additional 24 h in the presence or absence of J147 and then subjected to the MTT reduction assay. To assess knockdown efficiency, cells were harvested 48 h after transfection without subsequent stress exposure. For analysis of CHOP expression, cells were exposed to TM in the presence or absence of J147 for the final 6 h of the 48-h post-transfection period.

### 2.10. Statistical Analysis

All statistical analyses were performed using GraphPad Prism version 9.0. All data were derived from independent biological replicates, representing independent cell samples prepared on different days. Technical replicate wells within each independent experiment were averaged to yield one value for that biological replicate. Data are presented as the mean ± standard error of the mean (S.E.) or standard deviation (S.D.), as indicated in the figure legends. For comparisons between two groups, an unpaired two-tailed Student’s *t*-test was used. Comparisons among multiple groups were performed using one-way analysis of variance (ANOVA), followed by Tukey’s post hoc multiple-comparison test. A *p*-value of less than 0.05 was considered to indicate statistical significance.

## 3. Results

### 3.1. Curcumin, CNB-001, and J147 Protect HT22 Cells Against Glu-Induced Oxidative Cell Death

**Figure 1:**
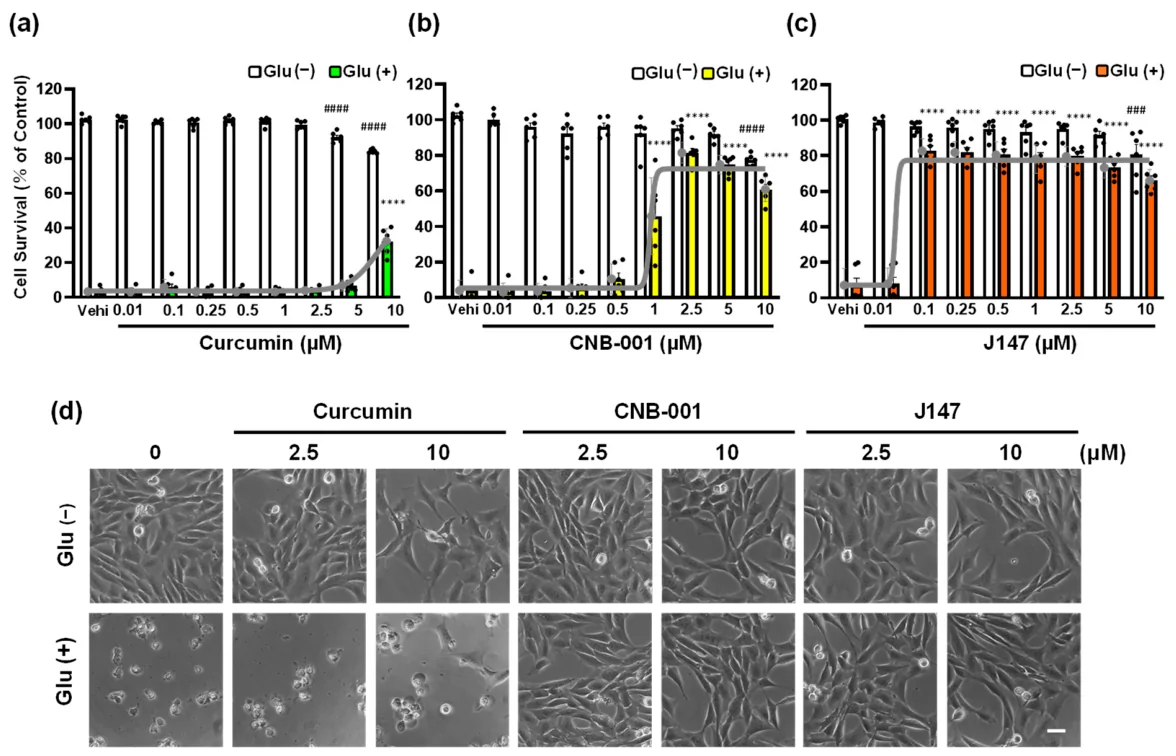
Neuroprotective effects of curcumin, CNB-001, and J147 against Glutamate (Glu)-induced oxidative cell death in HT22 cells. HT22 cells were treated with various concentrations of the indicated compounds in the presence or absence of 5 mM Glu for 24 h. Cell viability was measured using the MTT reduction assay and expressed as a percentage of untreated control cells. Graphs show the concentration-dependent effect of curcumin (**a**), CNB-001 (**b**), and J147 (**c**) on the MTT reduction activity and concentration–response curves of HT22 cells exposed to oxidative stress. (**d**) Photographs show typical phase-contrast microscopy images of vehicle-treated cells, Glu-treated cells, and cells co-treated with each compound. Data were derived from independent biological replicates, representing independent cell samples prepared on different days. Values represent means ± S.E. (*n* = 6). ^###^ *p* < 0.001, ^####^ *p* < 0.0001 vs. Vehicle, **** *p* < 0.0001 vs. Glu alone. Scale bar = 100 µm.

We first examined the effects of curcumin, CNB-001, and J147 on Glu-induced oxidative cell death in HT22 cells. Cytotoxicity experiments showed that curcumin reduced cell viability at ≥5 µM, whereas CNB-001 and J147 exhibited cytotoxicity at 10 µM (Figure 1). Therefore, subsequent experiments were performed within concentration ranges that did not cause overt cytotoxicity under basal conditions. The HT22 cells exposed to 5 mM Glu for 24 h had markedly lower viability than the vehicle-treated cells (Figure 1a–c). Co-treatment with curcumin, CNB-001, or J147 significantly attenuated this Glu-induced cytotoxicity in a concentration-dependent manner, but the effective concentration ranges differed among the compounds. Curcumin exerted significant protection only at 10 µM (Figure 1a), whereas CNB-001 was effective at ≥1 µM (Figure 1b). However, the apparent protective effects of curcumin and CNB-001 against Glu-induced cytotoxic conditions should be interpreted with caution because these compounds reduced basal MTT activity at higher concentrations. In contrast, J147 significantly preserved cell viability at concentrations as low as 0.1 µM (Figure 1c), indicating greater protective potency than curcumin or CNB-001 in this model.

The results of morphological assessment using phase-contrast microscopy were consistent with those of the MTT reduction assay. Consistent with cytotoxic injury, the vehicle-treated cells displayed normal cell morphology with extended cellular processes, whereas the Glu-treated cells exhibited marked shrinkage and rounding (Figure 1d). Curcumin partially preserved cell morphology, whereas CNB-001 and J147 appeared to preserve cell morphology more effectively. These results indicate that J147 potently protects HT22 cells from Glu-induced oxidative cell death, whereas the apparent protective effects of curcumin and CNB-001 were observed only at concentrations higher than those effective for J147.

### 3.2. J147 Selectively Protects HT22 Cells from Cell Death Under TM-Induced ER Stress

**Figure 2:**
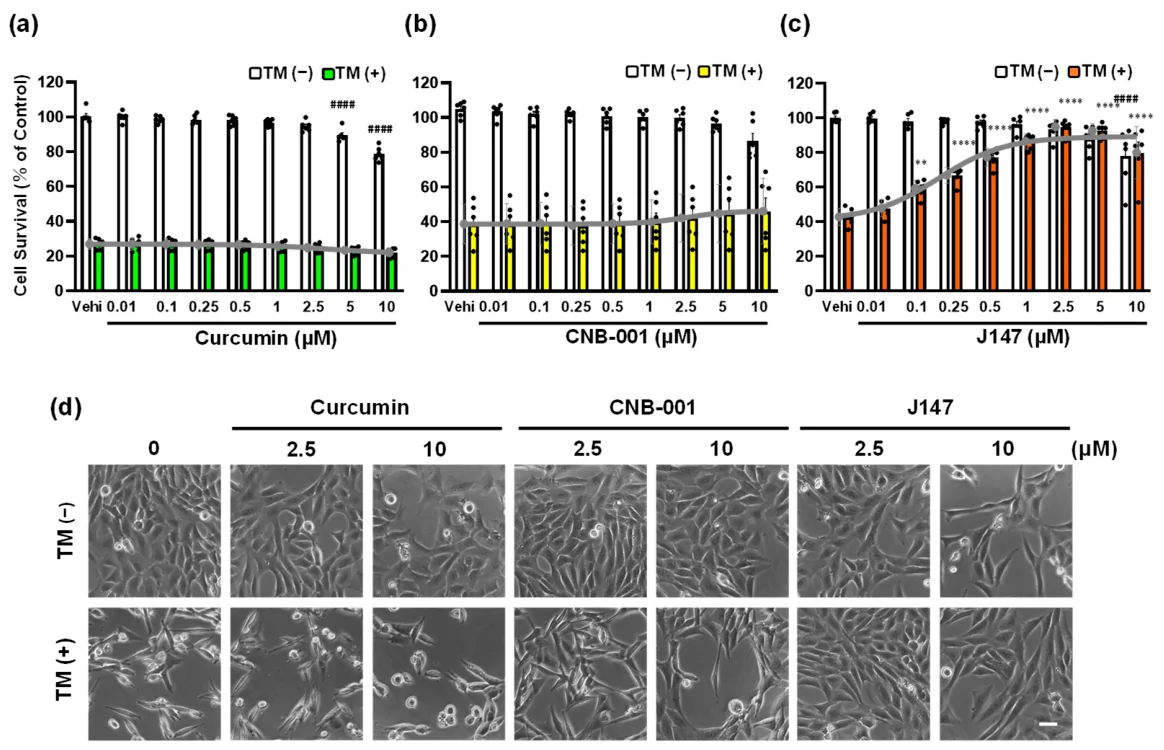
Selective protection by J147 against cell death under tunicamycin (TM)-induced endoplasmic reticulum (ER) stress conditions in HT22 cells. HT22 cells were treated with various concentrations of the indicated compounds in the presence or absence of 100 ng/mL TM for 24 h. Graphs show the concentration dependency of the effects of curcumin (**a**), CNB-001 (**b**), and J147 (**c**) on the MTT reduction activity and concentration–response curves of HT22 cells exposed to ER stress. Cell viability was measured using the MTT reduction assay and expressed as a percentage of untreated control cells. (**d**) Photographs show representative phase-contrast microscopy images of vehicle-treated cells, TM-treated cells, and cells co-treated with each compound. Data were derived from independent biological replicates, representing independent cell samples prepared on different days. Values represent means ± S.E. (*n* = 6). ^####^ *p* < 0.0001 vs. Vehicle, ** *p* < 0.01, **** *p* < 0.0001 vs. TM alone. Scale bar = 100 µm.

We investigated whether curcumin, CNB-001, or J147 can protect HT22 cells from ER stress-mediated cell death triggered by TM. The HT22 cells exposed to 100 ng/mL TM for 24 h had markedly lower viability than the vehicle-treated cells (Figure 2a–c). Neither curcumin nor CNB-001 attenuated TM-induced cytotoxicity at the concentrations tested (Figure 2a,b). In contrast, J147 significantly protected HT22 cells from TM-induced cell death in a concentration-dependent manner, with significant protection observed at concentrations as low as 0.1 μM (Figure 2c).

Phase-contrast microscopic observations supported these findings. The TM-treated cells showed pronounced morphological deterioration, including loss of cell-to-cell contacts and rounded or spindle-shaped morphology (Figure 2d). Curcumin and CNB-001 did not visibly prevent these morphological changes, whereas J147 largely preserved normal cell morphology and intercellular connectivity. These results indicate that among the three compounds tested, only J147 protects HT22 cells from cell death under TM-induced ER stress conditions.

### 3.3. Concentration–Response Analysis Indicates Greater Protective Potency of J147

The EC50 values of curcumin, CNB-001, and J147 were estimated from concentration-response curves under Glu- and TM-induced stress conditions to further compare their relative potencies (Figure 1a–c and Figure 2a–c). In the Glu-induced oxytosis model, the estimated EC50 value of J147 was 0.0327 µM, whereas those of CNB-001 and curcumin were 1.60 µM and >10 µM, respectively. Thus, J147 showed an approximately 50-fold lower EC50 than CNB-001, whereas the EC50 value of curcumin could not be precisely defined within the tested concentration range. In the TM-induced ER stress model, the EC50 value of curcumin could not be determined because of insufficient efficacy, and the estimated EC50 value of CNB-001 was >10 µM. In contrast, nonlinear regression of the J147 concentration–response curve yielded an estimated EC50 value of 0.259 µM for protection against TM-induced cytotoxicity. These results further support the higher potency of J147 in the Glu-induced oxidative stress model and its selective protection under TM-induced proteotoxic ER stress within the concentration range tested.

### 3.4. J147 Decreases EthD-1-Positive Dead Cells Under Oxidative and ER Stress Conditions

**Figure 3:**
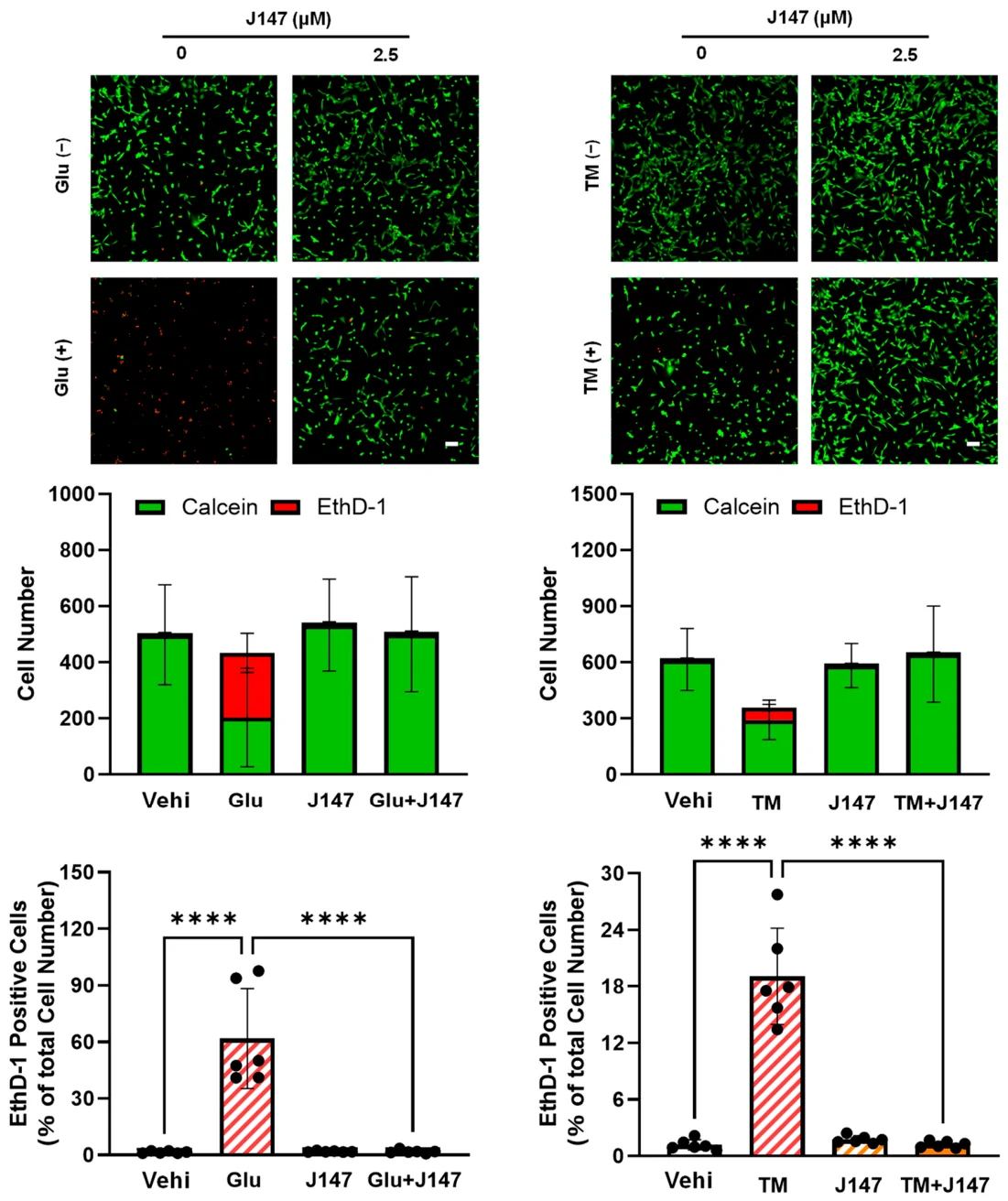
J147 reduces EthD-1-positive dead cells under oxidative and ER stress conditions. HT22 cells were exposed to either 5 mM Glu or 100 ng/mL TM for 24 h in the presence or absence of 2.5 µM J147. Representative fluorescence micrographs show LIVE/DEAD staining with calcein-AM (green, viable cells) and EthD-1 (red, dead cells) for each experimental group. The stacked bar graphs show the number of EthD-1-positive dead cells and calcein-AM-positive live cells in each experimental group. The lower graphs show the percentage of EthD-1-positive dead cells relative to the total number of cells. Data were derived from independent biological replicates, representing independent cell samples prepared on different days. Values represent means ± S.D. (*n* = 6). **** *p* < 0.0001. Scale bar = 100 µm.

We evaluated cell viability and death using LIVE/DEAD staining to further validate the cytoprotective effect of J147 (Figure 3). The HT22 cells exposed to 5 mM Glu or 100 ng/mL TM for 24 h had a markedly greater number of EthD-1-positive dead cells and a lower number of calcein-AM-positive viable cells than the vehicle-treated control cells (Figure 3). Conversely, co-treatment with 2.5 µM J147 substantially reduced the increase in EthD-1-positive dead cells induced by both Glu and TM while preserving calcein-AM-positive viable cells. Quantitative analysis confirmed that J147 significantly decreased the percentage of EthD-1-positive dead cells under both oxidative and ER stress conditions (Figure 3). These findings support the MTT reduction assay results and further demonstrate that J147 protects HT22 cells against both Glu-induced oxidative cell death and cell death under TM-induced ER stress conditions.

### 3.5. J147 Suppresses TM-Induced CHOP Expression Without Reducing GRP78 Induction

**Figure 4:**
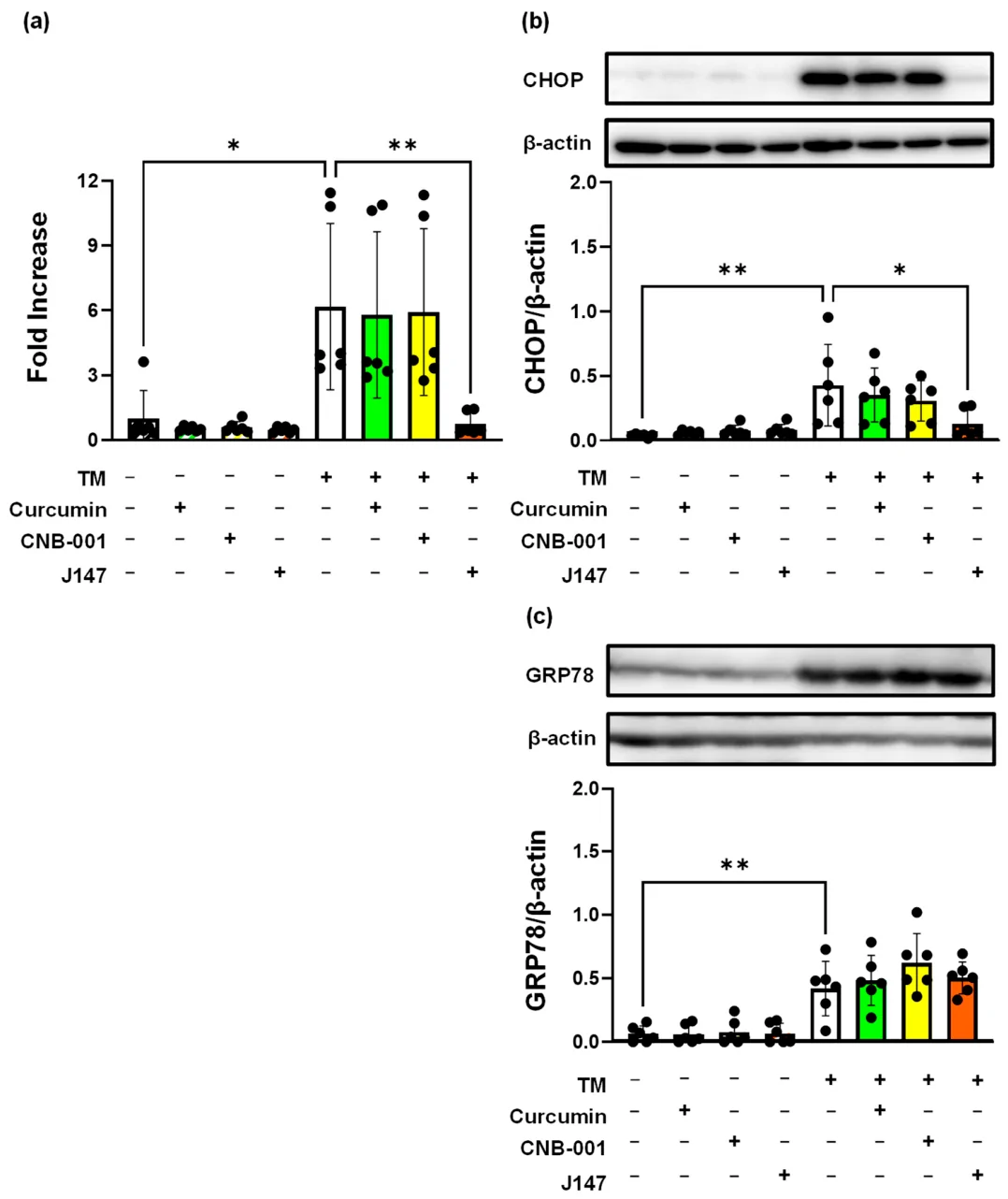
J147 suppresses TM-induced C/EBP homologous protein (CHOP) upregulation without inhibiting glucose-regulated protein 78 kDa (GRP78) induction. HT22 cells were treated with 100 ng/mL TM in the presence or absence of 2.5 µM curcumin, CNB-001, or J147. (**a**) Relative mRNA expression of Ddit3, which encodes CHOP, was quantified by real-time PCR after 3 h of treatment. (**b**) Representative western blot images (upper) and densitometric quantification (lower) of CHOP protein levels normalized to β-actin in cells treated for 6 h. (**c**) Representative western blot images (upper) and densitometric quantification (lower) of GRP78 protein levels normalized to β-actin in cells treated for 24 h. β-actin was used as an internal control. Data were derived from independent biological replicates, representing independent cell samples prepared on different days. Values represent means ± S.D. (*n* = 6). * *p* < 0.05, ** *p* < 0.01.

We next examined whether the protective effect of J147 against cell death under TM-induced ER stress conditions was associated with changes in ER stress signaling using real-time qPCR and western blot analysis. Exposure of HT22 cells to 100 ng/mL TM markedly increased the mRNA expression of Ddit3, which encodes CHOP, at 3 h (Figure 4a). Among the three compounds tested, only J147 significantly suppressed TM-induced Ddit3 upregulation, whereas curcumin and CNB-001 exerted no significant effects. Consistent with the qPCR results, western blot results showed that TM markedly increased CHOP protein expression at 6 h, and this induction was significantly attenuated by J147 but not by curcumin or CNB-001 (Figure 4b).

We then examined whether J147 also affects GRP78, an adaptive ER chaperone induced during the unfolded protein response. TM increased GRP78 protein expression at 24 h, whereas GRP78 induction remained evident in the presence of J147 (Figure 4c). Collectively, these results show that J147 reduced TM-induced CHOP expression, whereas TM-induced GRP78 expression remained evident in the presence of J147.

### 3.6. J147 Retains Its Protective Effect Under TM-Induced ER Stress After ATP5A Knockdown

**Figure 5:**
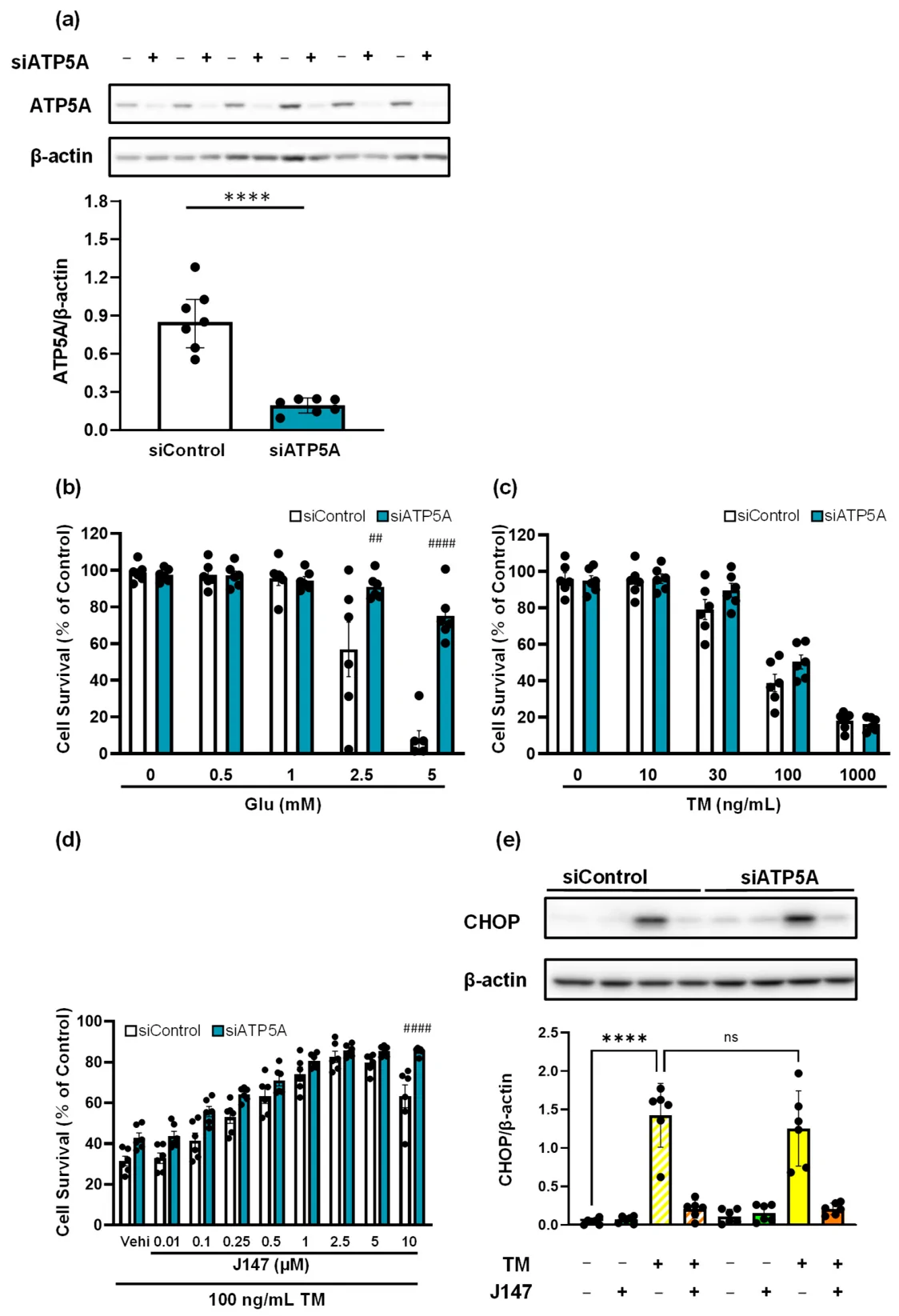
J147 retains its protective effect under TM-induced ER stress after ATP5A knockdown. (**a**) Western blot analysis confirming ATP5A knockdown efficiency. HT22 cells were transfected with control siRNA or ATP5A-specific siRNA, and protein lysates were analyzed 48 h after transfection. Control and ATP5A knockdown cells were exposed to Glu (0–5 mM) (**b**) or TM (0–1000 ng/mL) (**c**) for 24 h, and cell viability was measured by the MTT reduction assay. (**d**) Concentration-dependent effect of J147 on TM-induced cytotoxicity in control and ATP5A knockdown cells. HT22 cells were treated with various concentrations of J147 in the presence of 100 ng/mL TM for 24 h, and cell viability was assessed using the MTT reduction assay. (**e**) Representative western blot images (upper) and densitometric quantification (lower) of CHOP protein levels normalized to β-actin in control and ATP5A knockdown cells treated with J147 in the presence or absence of TM for 6 h. Data were derived from independent biological replicates, representing independent cell samples prepared on different days. Values represent the mean ± S.E. for MTT assays (*n* = 6) and the mean ± S.D. for western blot analysis (*n* = 6–7). ^##^ *p* < 0.01, ^####^ *p* < 0.0001 vs. siControl at the same concentration. **** *p* < 0.0001. ns: not significant.

J147 interacts with ATP5A, which is implicated in its protective effects against oxidative stress [26]. To determine whether ATP5A is also involved in the protective effect of J147 under TM-induced ER stress, we used ATP5A knockdown HT22 cells. Western blot analysis confirmed that ATP5A protein expression was markedly lower in the cells transfected with ATP5A-specific siRNA than in the cells transfected with control siRNA (Figure 5a).

We examined the effect of ATP5A knockdown on stress-induced cell death. In the Glu-induced oxidative stress model, ATP5A knockdown significantly attenuated the Glu-induced reduction in cell viability (Figure 5b). In contrast, ATP5A knockdown did not protect HT22 cells from TM-induced cytotoxicity (Figure 5c).

We then investigated whether J147 retains its protective effect against TM-induced cytotoxicity in ATP5A knockdown cells. J147 increased cell viability in both control and ATP5A knockdown cells exposed to TM (Figure 5d). Moreover, TM-induced CHOP protein expression was similarly suppressed by J147 in both control and ATP5A knockdown cells (Figure 5e). These findings support the involvement of ATP5A in Glu-induced oxidative cell injury and indicate that J147 can suppress TM-induced cytotoxicity and CHOP induction even after ATP5A knockdown.

## 4. Discussion

In the present study, we compared the protective effects of curcumin and its derivatives CNB-001 and J147 against Glu-induced oxytosis and TM-induced ER stress in HT22 hippocampal neuronal cells. Although all three compounds attenuated Glu-induced oxidative cell death, only J147 protected cells from death under TM-induced ER stress. This protection was accompanied by reduced Ddit3/CHOP induction, whereas TM-induced GRP78 expression remained evident in the presence of J147. However, these findings alone do not establish selective regulation of maladaptive and adaptive UPR signaling. Furthermore, although ATP5A knockdown reproduced the protective phenotype against oxidative injury, it neither prevented TM-induced cytotoxicity nor abolished the protective effect of J147. Together, these findings support at least partly distinct protective responses to oxidative and TM-induced stress, with ATP5A-associated protection in the Glu model and ATP5A-independent protection under TM exposure occurring in parallel with reduced CHOP induction.

J147 was originally identified through a curcumin-based phenotypic screening program designed to overcome the pharmacological limitations of curcumin, such as poor stability and bioavailability. This optimization process yielded a compound with significantly enhanced potency over both curcumin and CNB-001 in neuroprotection assays, particularly in oxidative stress and oxytosis models [21,22]. Consistent with this development background, J147 showed potent protection against Glu-induced oxidative cell death in the present study. The estimated EC50 of J147 was approximately 50-fold lower than that of CNB-001 in the Glu-induced oxytosis model. However, the apparent effect of curcumin at 10 µM should be interpreted cautiously because this concentration also reduced basal MTT-reducing activity. J147 also protected HT22 cells from cell death under TM-induced ER stress conditions, an effect not exerted by curcumin or CNB-001. TM induces ER stress by disrupting N-linked glycosylation and promoting misfolded protein accumulation in the ER [36,37], thereby imposing a form of proteotoxic stress that is mechanistically distinct from Glu-induced oxytosis. In support of this distinction, Shimazawa et al. demonstrated that N-acetylcysteine and edaravone protect SH-SY5Y cells against oxidative stress-related injury but not against TM-induced viability loss [45]. Moreover, although a previous study showed that curcumin can suppress CHOP induction under hypoxic stress [18], in the present study, this compound did not protect HT22 cells from TM-induced cell death. Therefore, the protective effect of J147 under TM-induced ER stress is unlikely to be attributable solely to its previously reported antioxidant or oxytosis-suppressing properties. Instead, J147 appears to possess an additional pharmacological action associated with reduced CHOP induction under TM-induced ER stress.

A key feature of J147 observed in this study was its preferential suppression of CHOP induction during TM-induced ER stress. In neuronal cells, excessive CHOP induction has been associated with ER stress-mediated cell death; our previous research showed that suppression of CHOP expression is accompanied by protection against ER stress-induced cytotoxicity in HT22 cells [33], organotypic rat hippocampal slice cultures, and primary cultured rat hippocampal neurons [46]. In addition, mithramycin A, a guanosine–cytosine-rich DNA-binding agent that inhibits Sp-family transcription factor binding, attenuates CHOP expression and protects hippocampal CA1 neurons from ischemia/reperfusion-induced injury in mice [47]. These findings support the idea that the pharmacological suppression of CHOP-associated signaling can contribute to neuronal protection under pathological stress. In the present study, J147 significantly reduced TM-induced Ddit3/CHOP expression at the mRNA and protein levels, whereas curcumin and CNB-001 did not. However, because CHOP was not genetically manipulated in the present study, these findings do not establish that reduced CHOP expression mediates J147-induced cytoprotection. Importantly, J147 did not suppress TM-induced expression of GRP78, an adaptive ER chaperone that contributes to protein folding and ER homeostasis restoration. Indeed, coordinating GRP78 and CHOP dynamics to restore UPR balance is a key therapeutic approach to mitigating ER stress injury in HT22 cells. For example, a sigma-1 receptor antagonist counters TM-induced cytotoxicity by driving ATF6-mediated GRP78 upregulation, thereby indirectly reducing CHOP levels [48]. In contrast, J147 reduced CHOP induction while TM-induced GRP78 expression remained detectable. However, evaluating these two markers alone does not establish selective regulation of adaptive and maladaptive UPR signaling or identify the upstream UPR branch affected by J147. Analyses of the PERK–eIF2α–ATF4, ATF6, and IRE1 pathways will be required to determine where J147 acts within the UPR. Previous studies have shown that caspase-3/7 activity increases only gradually after approximately 6 h of TM exposure, indicating delayed activation of the executioner caspases during ER stress [49]. Previous findings that executioner caspase activation occurs later during TM exposure are consistent with, but do not establish, an early site of action for J147. A previous study reported that J147 suppressed PERK-associated CHOP induction in a mouse model of traumatic brain injury [28]. However, the present data do not establish whether the PERK–eIF2α–ATF4, ATF6, IRE1, or another signaling pathway accounts for the reduction in CHOP expression observed in HT22 cells.

**Figure 6:**
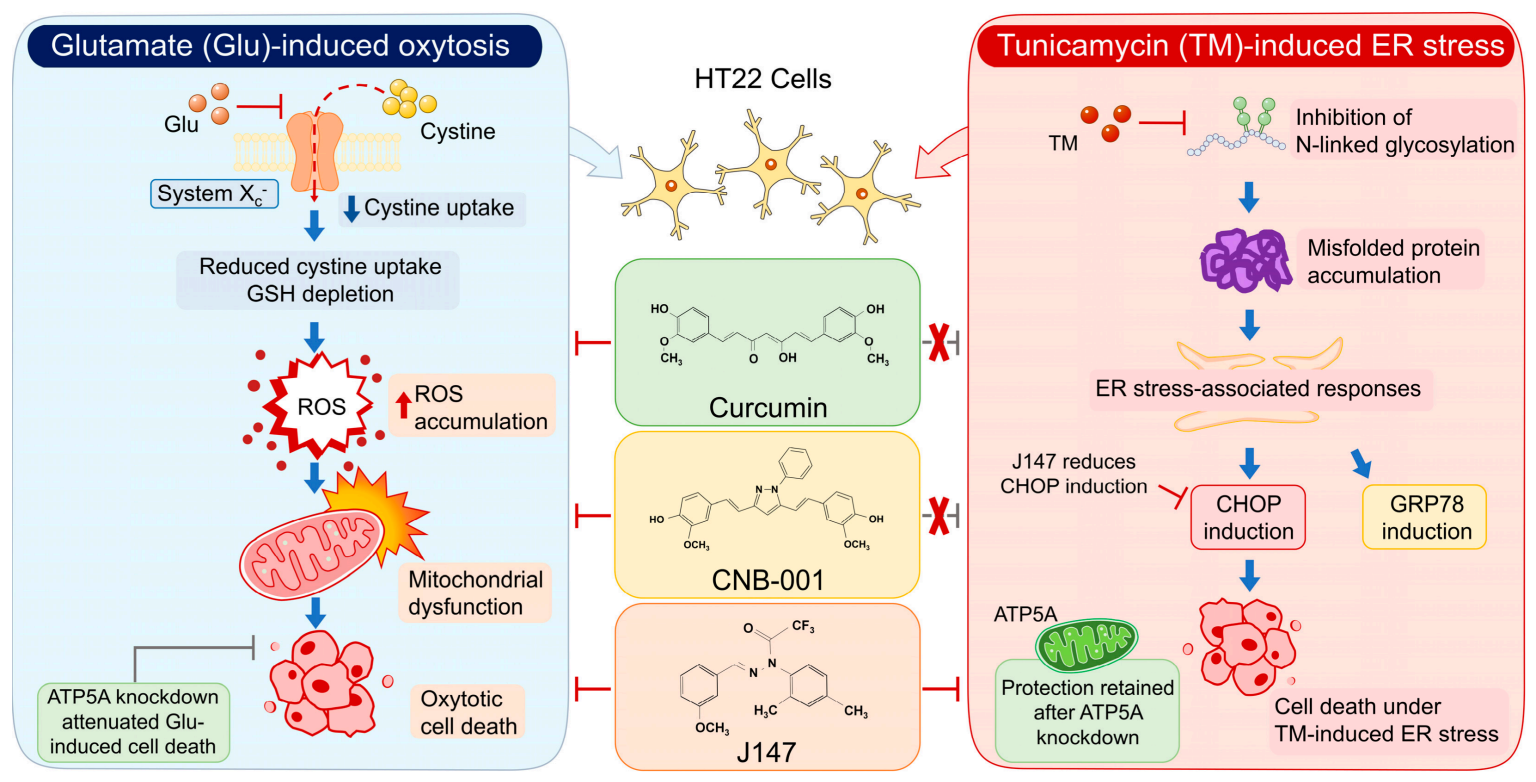
Schematic summary of the differential protective effects of curcumin, CNB-001, and J147 under Glu- and TM-induced stress in HT22 cells. Curcumin, CNB-001, and J147 attenuated Glu-induced oxytotic cell death, whereas only J147 protected cells from death under TM-induced ER stress. ATP5A knockdown attenuated Glu-induced cell death, but not cell death under TM-induced ER stress. Under TM-induced ER stress, J147-mediated protection was retained after ATP5A knockdown and occurred in parallel with reduced CHOP induction, whereas GRP78 induction remained evident.

J147 regulates mitochondrial function, cellular energy metabolism, and oxidative stress by targeting ATP5A [26]. Consistent with this previous model, the present study confirmed that ATP5A knockdown attenuated Glu-induced oxidative cell death in HT22 cells, reproducing the protective involvement of ATP5A-associated mitochondrial signaling against oxytosis. In contrast, ATP5A knockdown did not protect HT22 cells from TM-induced cytotoxicity, indicating that ATP5A suppression alone is insufficient to prevent chemically induced proteotoxic ER stress-induced cell death. These findings support the interpretation that the protective action of J147 against TM-induced proteotoxic ER stress contains an ATP5A-independent component. However, oxidative stress and ER stress are closely interconnected rather than completely independent processes. ER stress can alter cellular redox homeostasis and promote ROS generation, whereas oxidative stress can disrupt ER proteostasis and calcium homeostasis [3,27]. Emerging evidence in HT22 cells suggests that Nrf2-dependent antioxidant responses regulate cellular ROS levels during pharmacologically induced ER stress [50]. Thus, the differential effects of ATP5A knockdown observed in the present study may reflect stressor-dependent engagement of interconnected cellular stress networks rather than completely separate pathways. Because redox signaling was not directly examined in the present study, however, the molecular basis of the ATP5A-independent protective action of J147 under TM-induced ER stress remains to be determined. A schematic summary of the stress pathways examined and the principal findings of the present study are shown in Figure 6.

Oxidative stress, ER stress, mitochondrial dysfunction, and impaired proteostasis are interconnected cellular processes that have been implicated in neurodegenerative disorders [2,51]. Compounds capable of modulating more than one cellular stress response may, therefore, be useful pharmacological tools for investigating complex stress networks. J147 is relevant to this concept because it was originally identified through phenotypic screening designed to select compounds that are active against multiple age-associated cellular stress conditions, including oxidative stress, impaired energy metabolism, and amyloid toxicity [21,22]. J147 improves cognitive performance and preserves synaptic function in aged AD model mice [26] and rapidly aging SAMP8 mice [24]. It also modulates metabolic and mitochondrial features associated with brain aging [52]. However, the TM model used in the present study is a simplified pharmacological model of proteotoxic ER stress. Inhibition of N-linked glycosylation by TM does not reproduce the chronic accumulation of disease-specific protein aggregates, such as amyloid-β or α-synuclein, in the brain. Accordingly, the present findings should not be interpreted as direct evidence that J147 modifies the pathology or clinical course of AD, PD, or other protein aggregation disorders. Rather, the present study adds a cellular observation to the pharmacological profile of J147 by showing that it protects HT22 cells from cell death under TM-induced ER stress in parallel with reduced CHOP induction.

This study has certain limitations. First, HT22 cells are immortalized, proliferative cells and do not fully reproduce the biological properties of mature, post-mitotic hippocampal neurons. Because most compound-comparison and ATP5A-knockdown experiments relied on MTT-reducing activity, changes in this measure may reflect alterations in cell number, proliferation, and cellular metabolism in addition to cell death. Although LIVE/DEAD staining in selected experiments showed that J147 reduced the proportion of EthD-1-positive dead cells under both Glu- and TM-induced injury conditions, extrapolation of these findings to mature neurons requires caution. Validation in primary neuronal cultures, protein aggregation models, and disease-relevant in vivo models will be required before these findings can be related to neurodegenerative disease pathology. Second, although J147 reduced CHOP induction in parallel with cytoprotection, the present findings demonstrate an association rather than a causal relationship. It remains unclear whether reduced CHOP expression directly mediates the protective effect of J147 or occurs secondarily to the attenuation of upstream cellular stress. Genetic manipulation of CHOP, including knockdown or overexpression, will be required to determine its causal contribution to J147-mediated cytoprotection. Third, the upstream UPR pathways involved in the J147-associated reduction in CHOP expression were not investigated. Because only CHOP and GRP78 were evaluated, the present study cannot determine whether J147 differentially regulates adaptive and maladaptive UPR signaling. Activation of the PERK–eIF2α–ATF4, ATF6, and IRE1 pathways should be examined in future studies to identify the site of J147 action within the UPR. Fourth, ATP5A involvement was assessed using a single siRNA preparation without validation using an independent siRNA sequence or a genetic rescue experiment; therefore, residual ATP5A expression and potential off-target effects cannot be completely excluded. In addition, most compound comparisons and ATP5A-knockdown experiments relied on MTT-reducing activity, which reflects cellular metabolic activity as well as cell survival. Finally, TM was the only pharmacological ER stress inducer used in the present study. Although TM-induced increases in CHOP and GRP78 supported the activation of ER stress responses, it remains unknown whether J147 also protects against cell death induced by mechanistically distinct forms of ER stress. Therefore, the present findings cannot be directly extrapolated to the pathophysiology or treatment of AD, PD, or other protein aggregation disorders. Future studies using thapsigargin, which induces ER stress by disrupting ER calcium homeostasis, and other ER stressors will be required to determine the generalizability of the protective effect of J147.

## 5. Conclusions

In conclusion, J147, but not curcumin or CNB-001, protects HT22 hippocampal neuronal cells from TM-induced ER stress-associated cell death. This protective effect was accompanied by suppression of CHOP induction without reduction of GRP78 expression and was retained in ATP5A knockdown cells. In contrast, ATP5A knockdown attenuated Glu-induced oxidative cell injury, whereas it did not attenuate TM-induced cytotoxicity. These findings support the existence of at least partly distinct mechanisms underlying the protective actions of J147 against oxidative stress- and ER stress-induced cytotoxicity. In the Glu-induced oxytosis model, J147-related protection was associated with ATP5A-linked mechanisms. Under TM exposure, J147-mediated protection was retained after ATP5A knockdown and occurred in parallel with reduced CHOP induction. Further elucidation of this ATP5A-independent component of J147-mediated protection may provide new insight into the cellular regulation of stress responses.

## Data Availability

The original contributions presented in this study are included in the article and Supplementary Materials. Further inquiries can be directed to the corresponding author.

## Supplementary Materials

The following supporting information can be downloaded at https://www.mdpi.com/article/10.3390/cells15212005/s1. Supplementary File S1: Original uncropped Western blot images and raw numerical data underlying the figures.

## Abbreviations

The following abbreviations are used in this manuscript:

AD	Alzheimer’s disease
ALS	amyotrophic lateral sclerosis
ANOVA	analysis of variance
ATF4	activating transcription factor 4
ATF6	activating transcription factor 6
ATP5A	ATP synthase α-subunit
C/EBP	CCAAT/enhancer-binding protein
cDNA	complementary DNA
CHOP	C/EBP homologous protein
Ddit3	DNA damage-inducible transcript 3
DMSO	dimethyl sulfoxide
EC_50_	half-maximal effective concentration
ECL	enhanced chemiluminescence
eIF2α	eukaryotic initiation factor 2α
ER	endoplasmic reticulum
EthD-1	ethidium homodimer-1
GAPDH	glyceraldehyde-3-phosphate dehydrogenase
Glu	glutamate
GRP78	glucose-regulated protein 78
HBSS	Hanks’ balanced salt solution
IRE1	inositol-requiring enzyme 1
mRNA	messenger RNA
MTT	3-(4,5-dimethylthiazol-2-yl)-2,5-diphenyl tetrazolium bromide
Nrf2	nuclear factor erythroid 2-related factor 2
PD	Parkinson’s disease
PERK	protein kinase RNA-like ER kinase
qPCR	quantitative real-time PCR
ROS	reactive oxygen species
S.D.	standard deviation
SDS	sodium dodecyl sulfate
S.E.	standard error of the mean
siRNA	small interfering RNA
TM	tunicamycin
TTBS	Tris-buffered saline containing 0.05% Tween 20
UPR	unfolded protein response
