# Supplementary material for: Curcumin-Derived J147 Protects HT22 Cells from Cell Death Under Tunicamycin-Induced Endoplasmic Reticulum Stress with CHOP Suppression Retained After ATP5A Knockdown
Source: Cells. 2026 Nov 3;15(21):2005. doi: 10.3390/cells15212005 (PMC13762192; doi:10.3390/cells15212005)
Supplement: Supplementary file 1 [file cells-15-02005-s001.zip › cells-4636929_Supplementary_File_S1.zip!/README.pdf]

Numerical Data and Western Blot Image Overview (README)

The numerical data files and Western blot images described below are provided together in cells-4636929_Supplementary_File_S1.zip.

1. File Inventory & Figure Mapping

This dataset contains numerical values from independent experiments used for figure preparation and statistical analysis. The level of measurement and processing provided for each assay is specified in Section 7. In addition, all uncropped original Western blot image files—including all replicates not presented in the main text—are included directly in this single zip archive.

Curcumin_MTT raw data.xls

- Corresponding Figures: Fig. 1a (Glutamate toxicity) & Fig. 2a (Tunicamycin toxicity)
- Assay Type: MTT Cell Viability / Reduction Assay

CNB-001_MTT raw data.xlsx

- Corresponding Figures: Fig. 1b (Glutamate toxicity) & Fig. 2b (Tunicamycin toxicity)
- Assay Type: MTT Cell Viability / Reduction Assay

J147_MTT raw data.xls

- Corresponding Figures: Fig. 1c (Glutamate toxicity) & Fig. 2c (Tunicamycin toxicity)
- Assay Type: MTT Cell Viability / Reduction Assay

LIVEDEAD raw data.xlsx

- Corresponding Figures: Fig. 3 (Tunicamycin & Glutamate toxicity)
- Assay Type: LIVE/DEAD Cell Assay (Calcein AM / EthD-1 fluorescent cell counts)

J147_rt-qPCR raw data.xlsx

- Corresponding Figures: Fig. 4a (CHOP mRNA expression)
- Assay Type: Quantitative Real-Time PCR (rt-qPCR)

J147_WB raw data.xlsx

- Corresponding Figures: Fig. 4b (CHOP protein), Fig. 4c (GRP78 protein), Fig. 5a (ATP5A protein), Fig. 5e (CHOP protein)
- Assay Type: Western Blot Densitometric Quantification

siATP5A_MTT raw data.xlsx

- Corresponding Figures: Fig. 5b (Glutamate toxicity under siATP5A), Fig. 5c (Tunicamycin toxicity under siATP5A), & Fig. 5d (Protective effect of J147 against Tunicamycin under siControl/siATP5A)
- Assay Type: MTT Cell Viability / Reduction Assay

Western Blot Original Images

The archive contains uncropped original Western blot image files for all independent biological replicates (including unselected replicates). The specific image files representative of the cropped images displayed in the main figures are identified as follows:

Fig. 4B: Fig. 4b CHOP② and Fig. 4b beta-actin②

Fig. 4C: Fig. 4c GRP78① and Fig. 4c beta-actin①

Fig. 5A: Fig. 5A ATP5A and Fig. 5A beta-actin

Fig. 5E: Fig. 5e CHOP① and Fig. 5e beta-actin①

2. Measurement Units

MTT Assays (Fig. 1a–c, Fig. 2a–c, Fig.5b–d): Percentage of control.

LIVE/DEAD Assay (Fig. 3): Cell count per microscopic field (cells/field) and percentage of dead cells (% EthD-1).

rt-qPCR (Fig. 4a): Cycle threshold (Ct) values and relative Ddit3/CHOP mRNA abundance normalized to the vehicle control group.

Western Blot (Fig. 4b–c, Fig. 5a, Fig. 5e): Relative protein expression level (densitometric signal ratio normalized to internal loading control).

3. Identification of Independent Experiments

Sample identification numbers representing independent experiments are specified within the rows or columns of each raw data file. Technical replicate wells within the same experimental condition and independent experiment were averaged to yield one value per independent biological replicate.

4. Data Processing & Normalization

MTT Assays

For MTT assays, the following processing procedure was used:

1. Absorbance of cell-free blank wells was subtracted from the corresponding sample absorbance values.
2. Technical replicate wells within the same experimental condition and independent experiment were averaged.
3. The mean value for each treatment condition was normalized to the designated control group, which was set to 100%.

4. The resulting normalized values were used for statistical analysis and figure preparation.

LIVE/DEAD

Cell numbers and dead-cell percentages were calculated from the corresponding microscopic fields. No additional normalization or mathematical transformation was applied unless explicitly indicated in the corresponding raw data file.

rt-qPCR

qPCR reactions were performed in duplicate. ΔCt values were calculated by subtracting Ct(GAPDH) from Ct(Ddit3/CHOP). The 2^(-ΔCt) values were calculated for each reaction, averaged for each sample, and normalized to the mean value of the TM-untreated vehicle control group, which was set to 1.0. GAPDH expression was confirmed to be stable across experimental conditions. Primer specificity was confirmed by melting-curve analysis.

Western Blot

For Western blot analysis, densitometric values were obtained from the corresponding protein bands. Background correction was performed before normalization. Target protein band intensity was divided by the intensity of the corresponding internal loading control from the same sample. The resulting normalized target/loading-control ratio was used as the final quantitative value for statistical analysis.
The original Western blot images are retained separately

5. Statistical Analysis

Statistical analyses were performed using GraphPad Prism version 9.0. Technical replicate wells were averaged to obtain one value per independent biological replicate. Comparisons between two groups were performed using an unpaired two-tailed Student's t-test. Multiple-group comparisons were performed using one-way ANOVA followed by Tukey's multiple-comparison test. A p-value < 0.05 was considered statistically significant.

EC50 Calculation

Concentration–response curves were fitted using a four-parameter logistic model with a variable slope in GraphPad Prism 9.0. EC50 values and 95% confidence intervals were estimated from the fitted curves. EC50 values were considered not estimable when a well-defined maximal response was not obtained within the tested concentration range.

6. Excluded Data and Analysis-Specific Data Selection

No independent biological/experimental replicate was excluded from the dataset because of the experimental result. The selection of concentrations included in individual statistical analyses does not represent exclusion of independent biological replicates.

7. Relationship Between Raw Data and Processed Data

The MTT files provide normalized percentages for independent experiments; the LIVE/DEAD file provides cell counts and dead-cell percentages; the rt-qPCR file provides Ct values and relative mRNA abundance; and the Western blot file provides normalized target/loading-control ratios. These values support the figure summaries and statistical analyses.

Well-level absorbance and blank measurements for the MTT assays, and band/background/loading-control intensity measurements preceding the reported Western blot ratios, are not included in this archive.
